# Supplementary material for: Structural and Biochemical Analyses of the Butanol Dehydrogenase from Fusobacterium nucleatum
Source: Int J Mol Sci. 2023 Feb 3;24(3):2994. doi: 10.3390/ijms24032994 (PMC9917632; doi:10.3390/ijms24032994)
Supplement: Supplementary file 1 [file ijms-24-02994-s001.zip › [IJMS]FnYqdH-Supplementary materials-Revised-Final.pdf]

Supplementary Data

Structural and biochemical analyses of the butanol dehydrogenase from *Fusobacterium nucleatum*

Xue Bai [1,2,†], Jing Lan [1,2,†], Shanru He [1,2], Tingting Bu [1,2], Jie Zhang [1,2], Lulu Wang [1,2,3], Xiaoling Jin [1,2,] Yuanchao Mao [1,2], Wanting Guan [1,2], Liying Zhang [1,2], Ming Lu [4], Hai-long Piao [5], Inseong Jo [6], Nam-Chul Ha [7], Chunshan Quan [1,2], Ki Hyun Nam [8,9,*] and Yongbin Xu [1,2,*]

[1] Department of Bioengineering, College of Life Science, Dalian Minzu University, Dalian 116600, Liaoning, China

[2] Key Laboratory of Biotechnology and Bioresources Utilization of Ministry of Education, College of Life Science, Dalian Minzu University, China

[3] School of Life Science and Biotechnology, Dalian University of Technology, No. 2 Linggong Road, Dalian 116024, Liaoning, China

[4] Shandong Provincial Key Laboratory of Energy Genetics, key Laboratory of Biofuel, Qingdao Institute of Bioenergy and Bioprocess Technology, Chinese Academy of Sciences, Qingdao 266101, Shandong, China

[5] CAS Key Laboratory of Separation Science for Analytical Chemistry, Dalian Institute of Chemical Physics, Chinese Academy of Sciences, Dalian 116023, Liaoning, China

[6] Infectious Diseases Therapeutic Research Center, Korea Research Institute of Chemical Technology, Daejeon 34114, Republic of Korea

[7] Department of Agricultural Biotechnology, College of Agriculture and Life Sciences, Seoul National University, Gwanak-gu, Seoul 00826, Republic of Korea

[8] Department of Life Science, Pohang University of Science and Technology, 35398 Pohang, Republic of Korea

[9] POSTECH Biotech Center, Pohang University of Science and Technology, 35398 Pohang, Republic of Korea

[*]Correspondence: structures@postech.ac.kr (K.H.N.); yongbinxu@dlnu.edu.cn (Y.X.)

[†] Both authors contributed equally to this work.

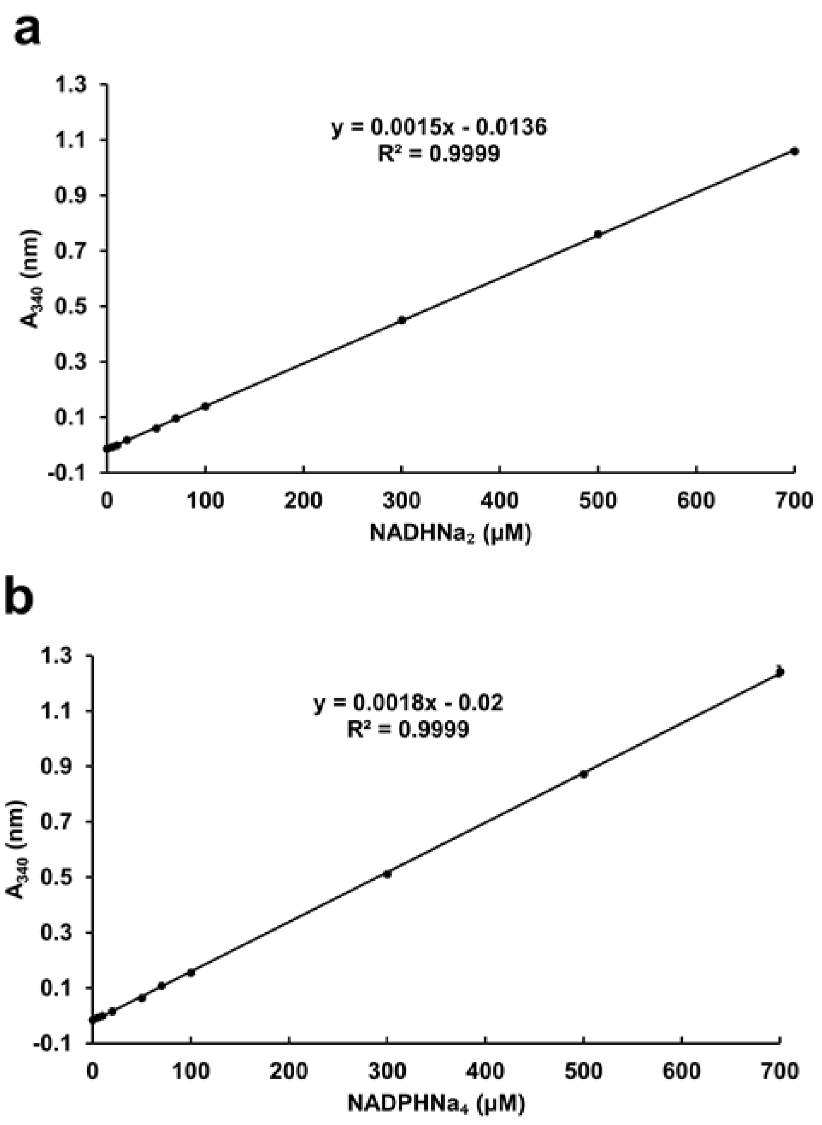

Figure S1. Calibration working curve constructed from the average slopes of (a) NADH, and (b) NADPH standards (0-700 μM), obtained from the three-fold assay repetition.

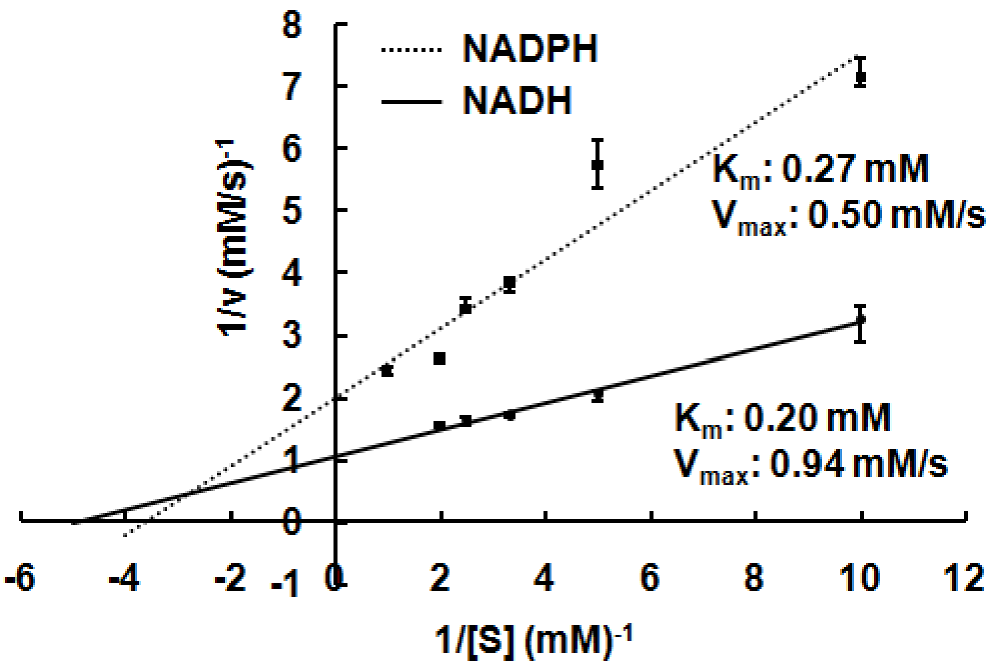

Figure S2. The double reciprocal plot was used to calculate V_{max} and K_m of the enzyme in the presence of NADH and NADPH. Data represent average ± SD of three different samples.

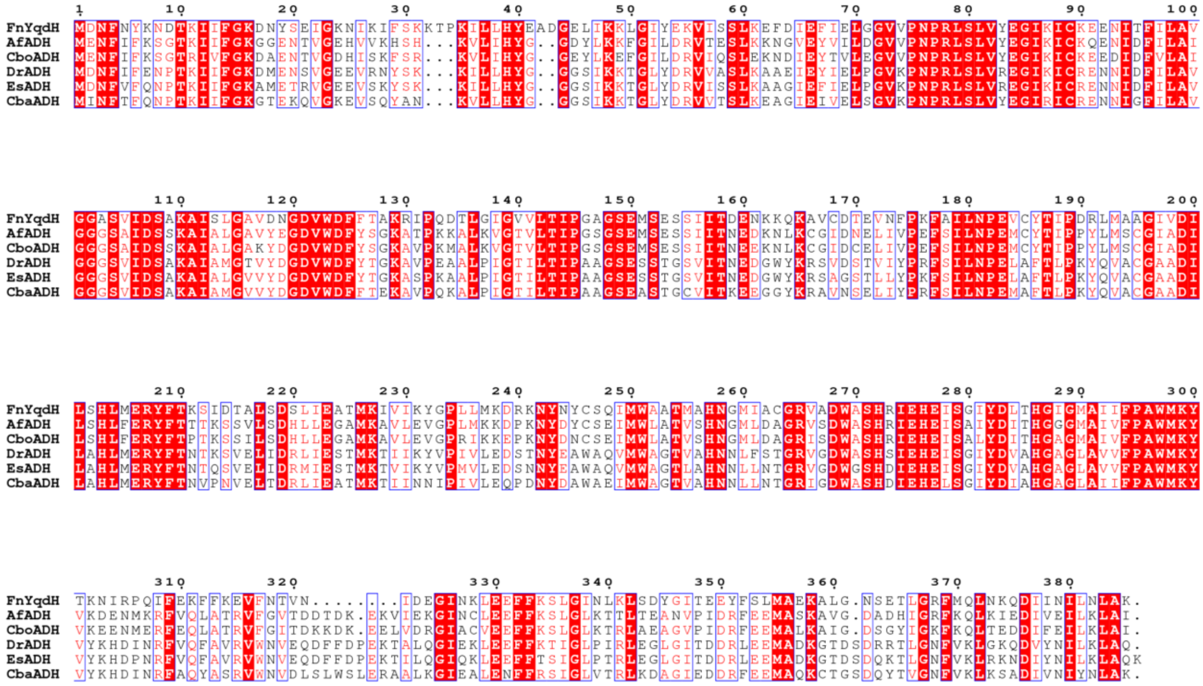

Figure S3. Sequence alignment of FnYqdH and homologous proteins**.** Sequence alignment of FnYqdH, AfADH (NCBI Reference Sequence: WP_066501503.1), CboADH (GenBank: QGU94363.1), DrADH (NCBI Reference Sequence: WSLF01000004), EsADH (GenBank: NLK98329.1) and CbaADH (GenBank: NLY43117.1)

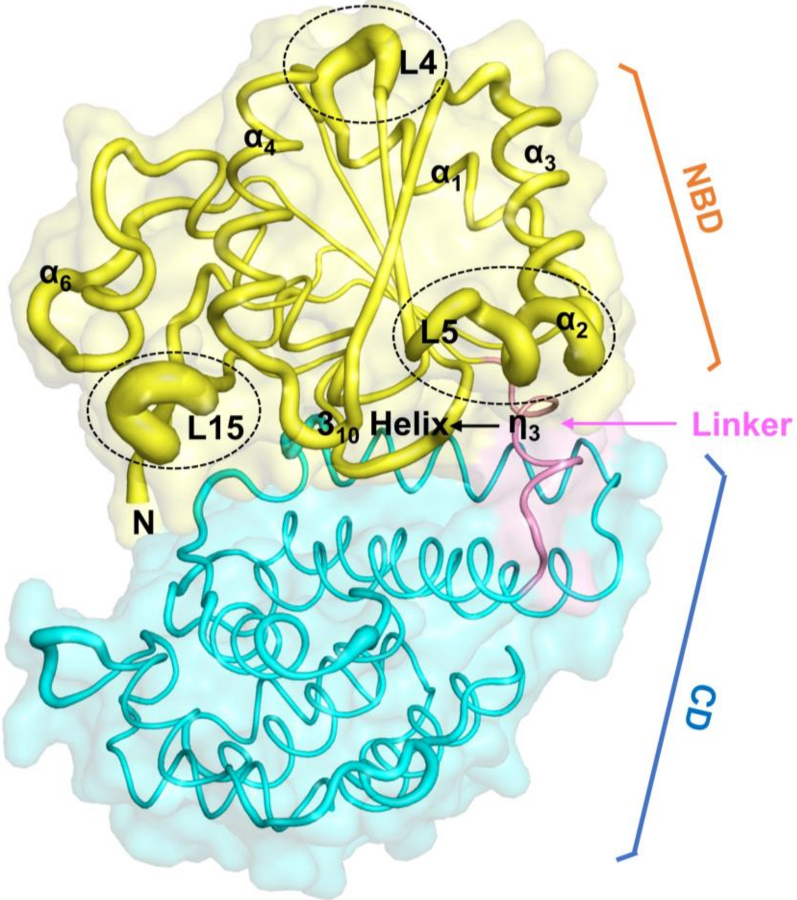

Figure S4. Analysis of the flexible region of FnYqdH. B-factor representation of FnYqdH; α2, L4, L5, and L15 show relatively high flexibility.

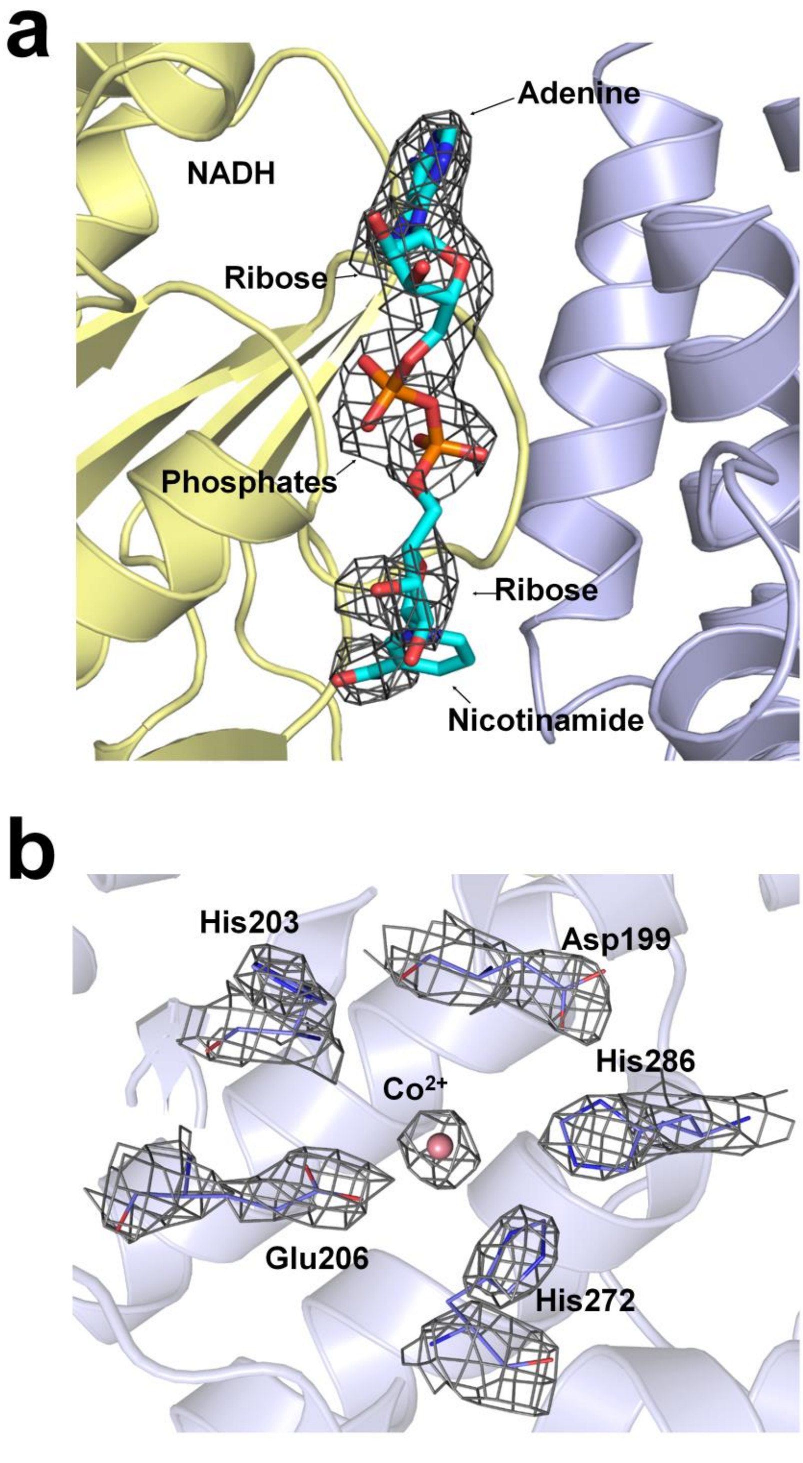

Figure S5. The electron density map of NADH and Co^{2+}. The electron density map of (a) NADH and (b) Co^{2+} are shown as grey mesh.

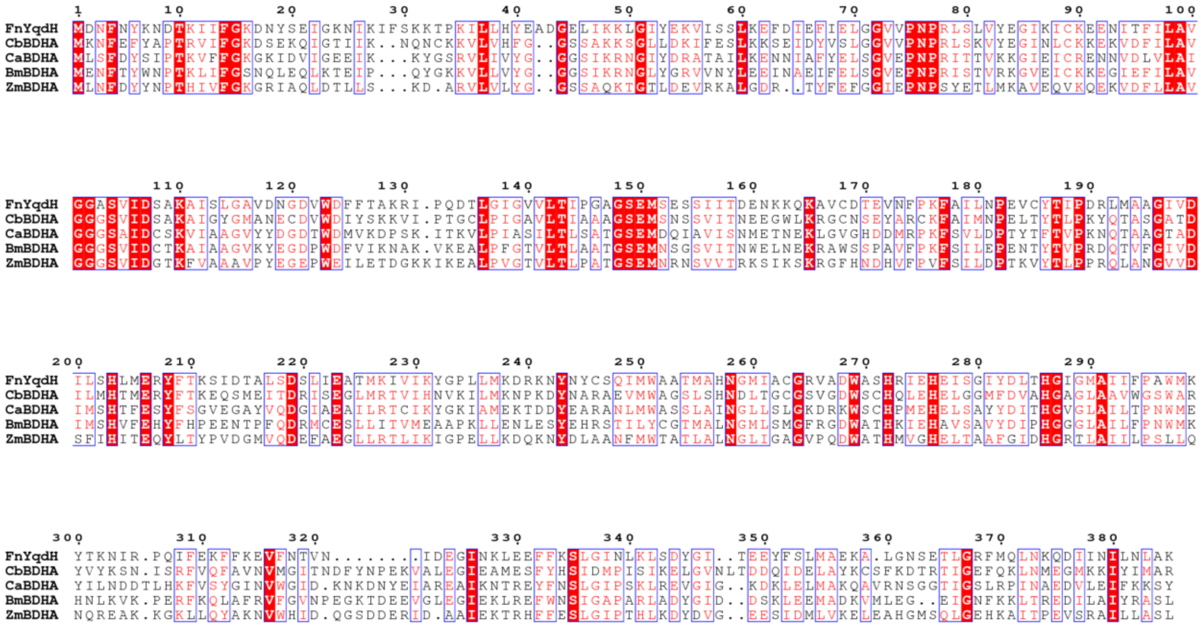

Figure S6. Sequence alignment of FnYqdH with BHD from *C. beijerinkii* (CbBDH, Accession No.: WP_077844128.1), *C. acetobutylicum* (CaBDHA, WP_010966572.1), *Bacillus megaterium* (BmBDHA, WP_013085305), and *Zymomonas mobilis* (ZmBDHA, WP_011241511.1).

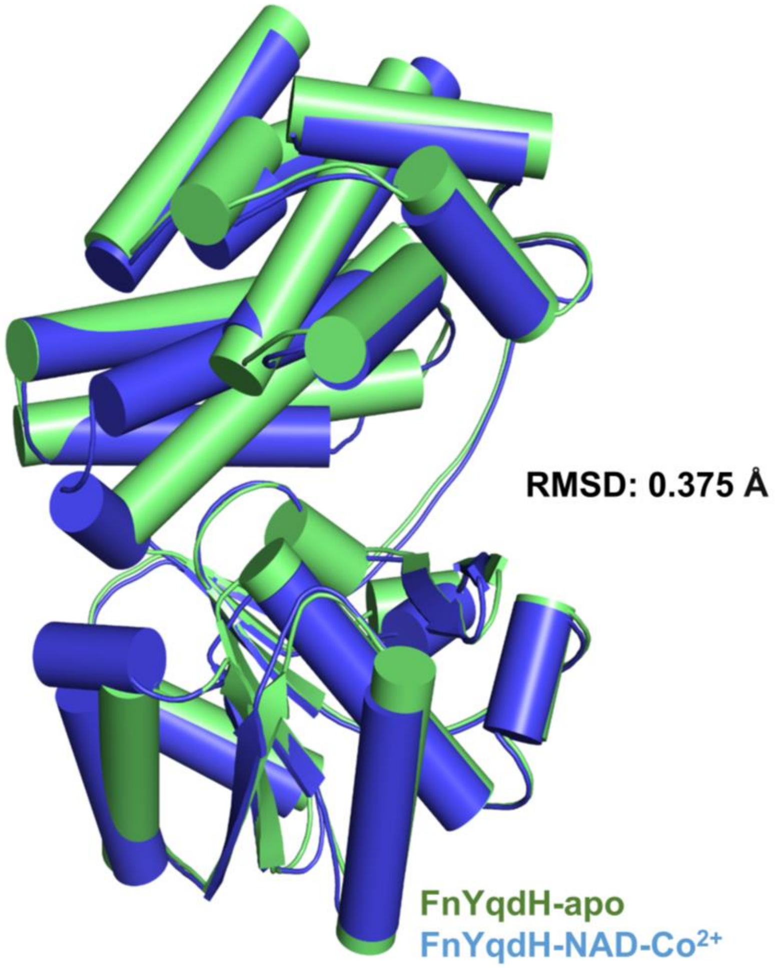

Figure S7. Overall fold of FnYqdH-apo and FnYqdH-NAD-Co^{2+}. Comparison of the structures of the FnYqdH-apo and FnYqdH-NAD-Co^{2+}. The crystal structures of the FnYqdH-apo and FnYqdH-NAD-Co^{2+} are colored green and blue, respectively.

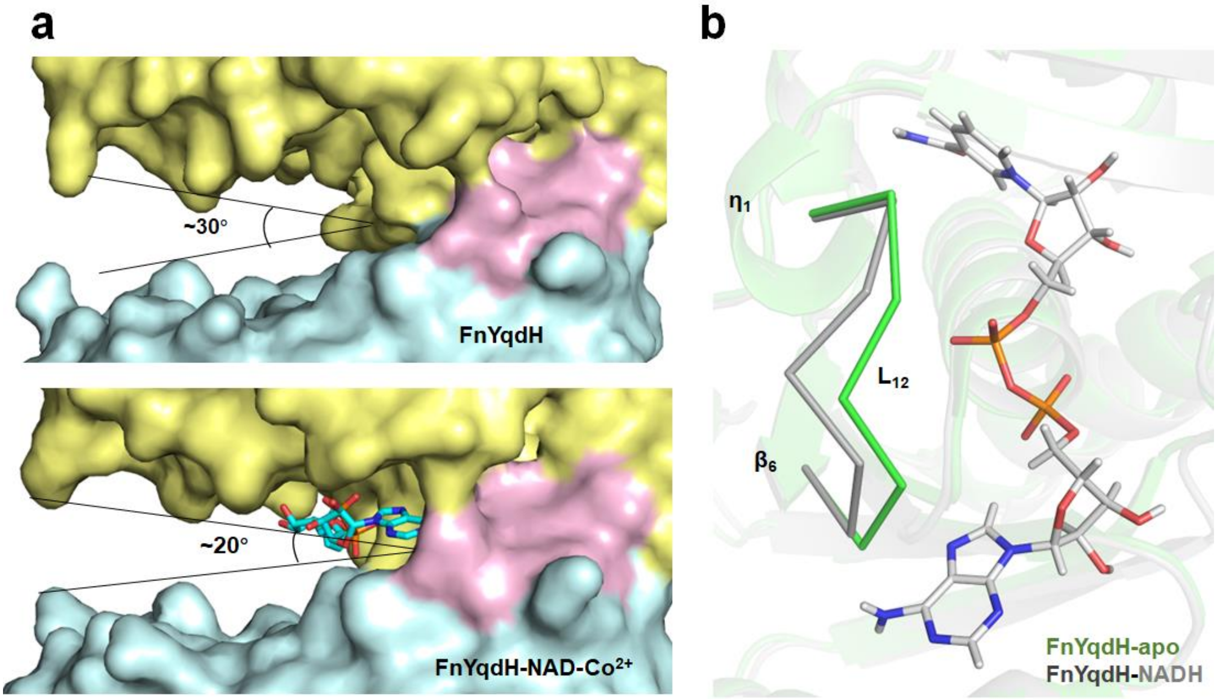

Figure S8. Comparison of FnYqdH-apo and FnYqdH-NAD-Co^{2+}. (a) Surface comparison of FnYqdH-apo and FnYqdH-NAD-Co^{2+}. (b) Cartoon compilation of FnYqdH-apo and FnYqdH-NAD-Co^{2+}. FnYqdH-apo and FnYqdH-NAD-Co^{2+} are colored green and grey, respectively.

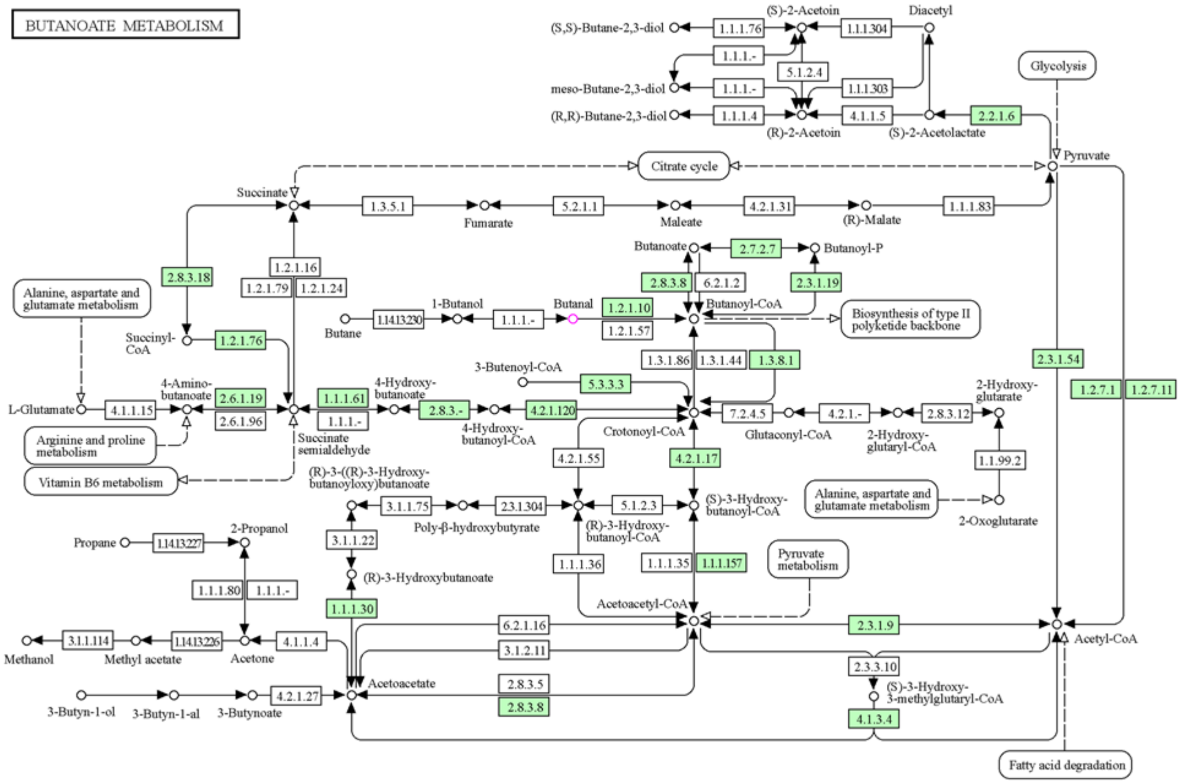

Figure S9. Butanoate metabolism of *Clostridioides difficile*.

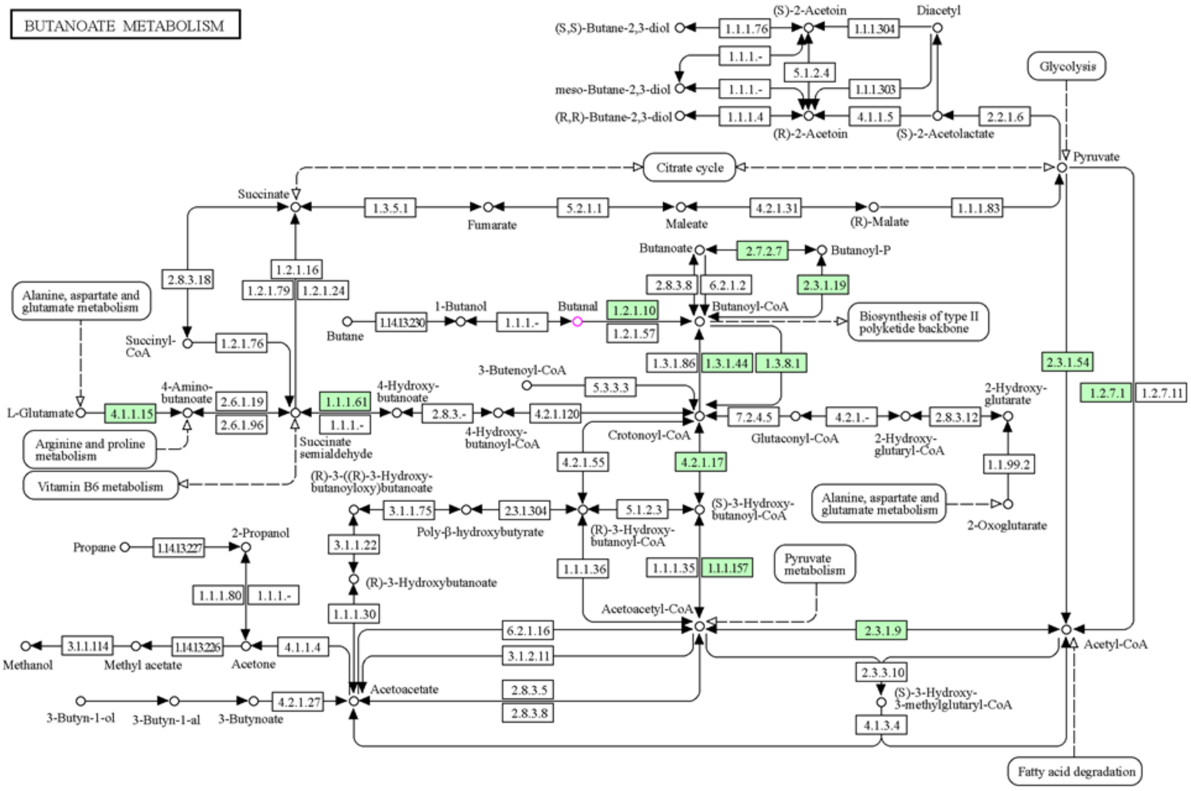

Figure S10. Butanoate metabolism of *Clostridium perfringens*.

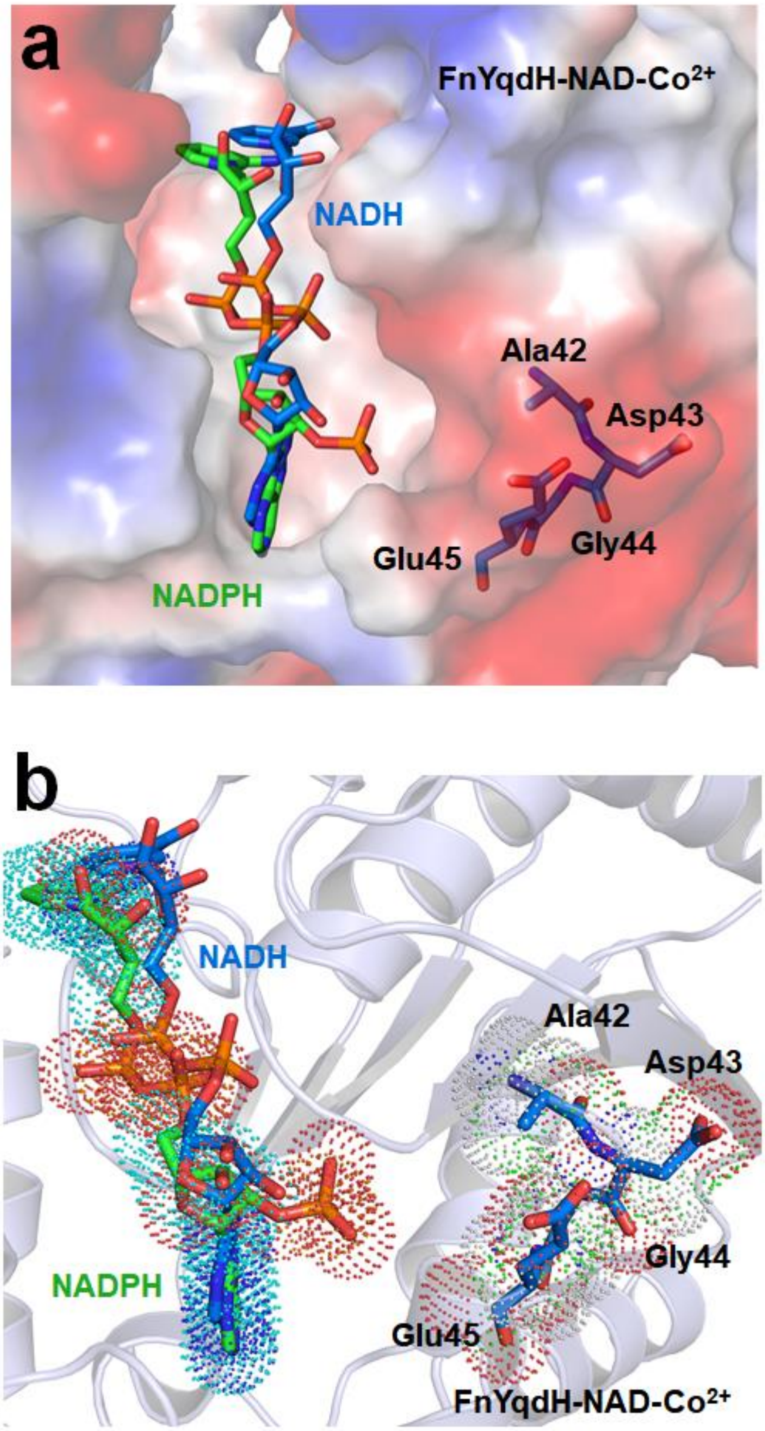

Figure S11. (a) The surface structure of the NAD(P)H-binding pocket of FnYqdH-NAD-Co2+, NADH is colored blue and NADPH is colored green. (b) Spatial distribution of NAD(P)H-binding pocket and NAD(P)H.

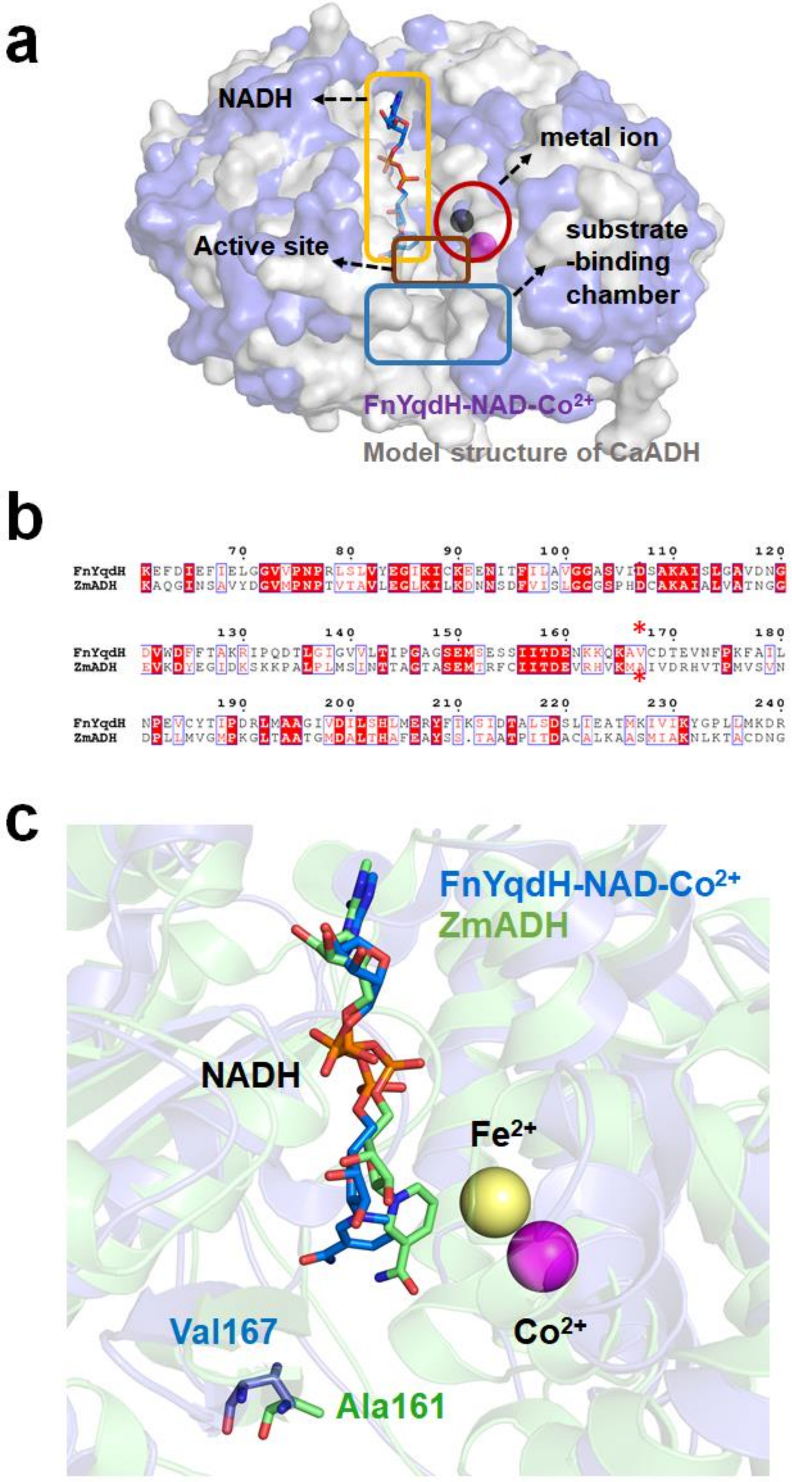

Figure S12. Comparison of FnYqdH-NAD-Co^{2+} and other structures. (a) Schematic representation of FnYqdH-NAD-Co2+ (colored blue) and CaADH (model structure, colored grey). (b) Partial sequence alignment of FnYqdH and ZmADH. The substrate-binding site of ZmBDH is indicated by a red *. (c)Comparison of FnYqdH-NAD-$^{Co2+}$ (colored marine [blue]) and ZmADH (colored green).

Table S1. Sequence of Primers.

Primers	Sequence
F-FnYqdH-WT	GGGCCATGGATATGGACAATTTTAATTATAAAAATGATAC
R-FnYqdH-WT	GGGCTCGAGTTATTTTGCTAAATTTAAGATA
F-FnYqdH-E41A	CTCTTGCATTATGCAGCAGATGGAGAA
R-FnYqdH-E41A	TTCTCCATCTGCTGCATAATGCAAGAG
F-FnYqdH-G44A	ATGAGGCAGATGCAGAATTAATAA
R-FnYqdH-G44A	TTATTAATTCTGCATCTGCCTCAT
F-FnYqdH-L46A	GCAGATGGAGAAGCAATAAAAAAACTT
R-FnYqdH-L46A	AAGTTTTTTTATTGCTTCTCCATCTGC
F-FnYqdH-S104A	GTTGGTGGAGCAGCAGTTATTGACTCA
R-FnYqdH-S104A	TGAGTCAATAACTGCTGCTCCACCAAC
F-FnYqdH-D107A	GCAAGTGTTATTGCATCAGCTAAAGCA
R-FnYqdH-D107A	TGCTTTAGCTGATGCAATAACACTTGC
F-FnYqdH-T143A	GGTTGTTTTAGCAATTCCAGGAGCT
R-FnYqdH-T143A	AGCTCCTGGAATTGCTAAAACAACC
F-FnYqdH-S152A	GGTTCTGAAATGGCAGAAAGCTCTATT
R-FnYqdH-S152A	AATAGAGCTTTCTGCCATTTCAGAACC
F-FnYqdH-K165A	AATAAGAAACAAGCAGCTGTTTGTGAT
R-FnYqdH-K165A	ATCACAAACAGCTGCTTGTTTCTTATT
F-FnYqdH-T187A	GAAGTTTGTTATGCAATTCCTGATAGA
R-FnYqdH-T187A	TCTATCAGGAATTGCATAACAAACTTC
F-FnYqdH-D199A	GCTGGAATTGTAGCAATTTTATCACAT
R-FnYqdH-D199A	ATGTGATAAAATTGCTACAATTCCAGC
F-FnYqdH-H203A	GATATTTTATCAGCATTAATGGAAAGA
R-FnYqdH-H203A	TCTTTCCATTAATGCTGATAAAATATC
F-FnYqdH-E206A	TCACATTTAATGGCAAGATATTTTACA
R-FnYqdH-E206A	TGTAAAATATCTTGCCATTAAATGTGA
F-FnYqdH-H272A	GATTGGGCTTCCGCAAGAATTGAACAT
R-FnYqdH-H272A	ATGTTCAATTCTTGCGGAAGCCCAATC
F-FnYqdH-H286A	TATGATTTGACTGCAGGTATTGGTATG
R-FnYqdH-H286A	CATACCAATACCTGCAGTCAAATCATA

The *Nco*I and *Xho*I restriction sites are underlined. The mutant sites are marked red color with underlined.
